# The replication machinery of LUCA: common origin of DNA replication and transcription

**DOI:** 10.1186/s12915-020-00800-9

**Published:** 2020-06-09

**Authors:** Eugene V. Koonin, Mart Krupovic, Sonoko Ishino, Yoshizumi Ishino

**Affiliations:** 1 National Center for Biotechnology Information, National Library of Medicine, National Institutes of Health, Bethesda, MD 20892 USA; 2 Archaeal Virology Unit, Institut Pasteur, 75015 Paris, France; 3 Department of Bioscience and Biotechnology, Graduate School of Bioresource and Bioenvironmental Sciences, Kyushu University, Fukuoka, 819-0395 Japan

## Abstract

Origin of DNA replication is an enigma because the replicative DNA polymerases (DNAPs) are not homologous among the three domains of life, Bacteria, Archaea, and Eukarya. The homology between the archaeal replicative DNAP (PolD) and the large subunits of the universal RNA polymerase (RNAP) responsible for transcription suggests a parsimonious evolutionary scenario. Under this model, RNAPs and replicative DNAPs evolved from a common ancestor that functioned as an RNA-dependent RNA polymerase in the RNA-protein world that predated the advent of DNA replication. The replicative DNAP of the Last Universal Cellular Ancestor (LUCA) would be the ancestor of the archaeal PolD.

## Introduction

DNA replication is a central process for all living cells [1]. Therefore, it is astonishing that the key enzymes involved in DNA replication, in particular, the replicative DNA polymerases (rDNAP), are unrelated among the 3 domains of life, Bacteria, Archaea, and Eukarya [2, 3]. This diversity of the replication machineries sharply contrasts with the conservation of the proteins involved in the other key processes of information transfer, namely, transcription and translation, as well as some key metabolic processes, such as nucleotide biosynthesis [4]. The lack of conservation of the rDNAPs and some other key components of the replication machinery, such as helicases and primases, complicates the reconstruction of the replicative apparatus of the ancestral life forms. Moreover, the existence of DNA genomes and, accordingly, DNA replication in the LUCA have been questioned, and the possibility of an RNA-based LUCA has been considered [2, 5]. Here, we focus on the nature of the DNA replication machinery of LUCA and, particularly, the rDNAP and the major evolutionary events that led to the drastic transformation of this machinery in the three domains of life and in viruses.

**Fig. 1.**
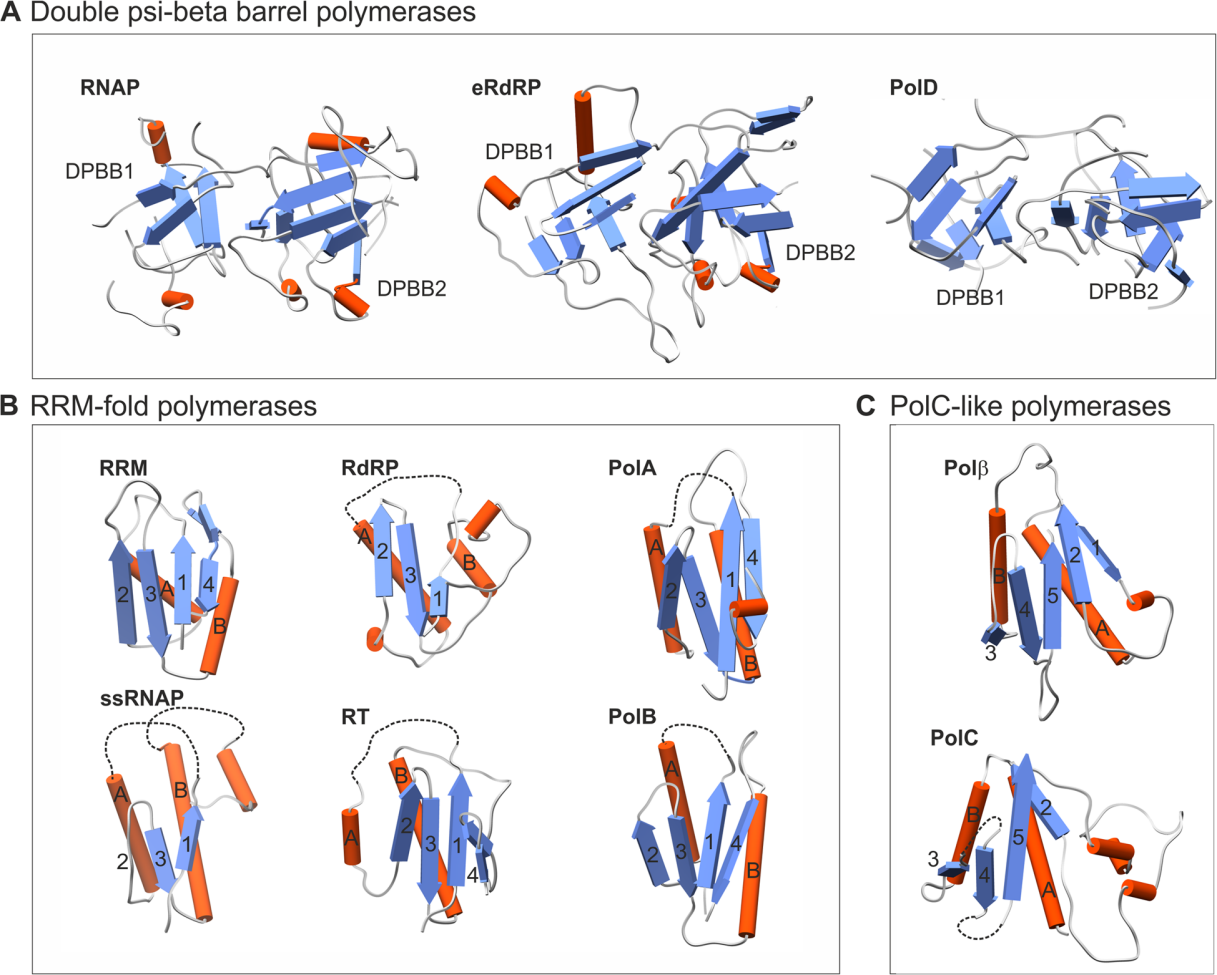
The core catalytic domains of DNA and RNA polymerases. **a** Double-psi beta-barrel (DPBB) polymerases. RNAP, multisubunit DNA-dependent RNA polymerase from *Thermus thermophilus* (PDB ID: 1iw7); eRdRP, eukaryotic RNA-dependent RNA polymerase from *Neurospora crassa* (PDB ID: 2j7n); PolD, DP2 subunit of family D DNA polymerase from *Pyrococcus abyssi* (PDB ID: 5ijl). **b** RRM-fold polymerases. RRM, RNA-recognition motif-containing RNA-binding domain of human nucleolysin TIAR (PDB ID: 2cqi); RdRP, RNA-dependent RNA polymerase of poliovirus type 1 (PDB ID: 1ra7); PolA, family A DNA polymerase from *Thermus aquaticus* (PDB ID: 1taq); ssRNAP, single-subunit DNA-dependent RNA polymerase of bacteriophage T7 (PDB ID: 1msw); RT, reverse transcriptase of Moloney murine leukemia virus (PDB ID: 1mml); PolB, family B DNA polymerase from *Thermococcus gorgonarius* (PDB ID: 1tgo). **c** PolC-like polymerases. Polβ, DNA polymerase β from *Rattus norvegicus* (PDB ID: 1bpb); PolC, family C DNA polymerase from *Thermus aquaticus* (PDB ID: 2hpi). In **b** and **c**, the major secondary structure elements forming the core palm domain are indicated with numbers (for β-strands) and capital letters (for α-helices). Dashed lines indicate regions where insertions into the core palm domains have occurred; these have been omitted for visualization purposes

There are several families of DNAPs that are involved in replication, repair, or both types of processes [6–8]. The replicative DNAPs of bacteria, archaea, and eukaryotes belong to 3 distinct protein families, and the core catalytic domains of these 3 DNAPs are unrelated to each other, i.e., adopt different protein folds as their catalytic cores (Fig. 1) and therefore are unlikely to share common ancestry (Table 1). The great majority of dsDNA viruses that infect either prokaryotes or eukaryotes and encode their own rDNAPs have the B family polymerase (PolB) that is also responsible for the replication in eukaryotes [9] (Table 1). Archaea encode multiple PolB copies, and with the exception of members of the order Crenarchaeota and some thermophilic members of the Thaumarchaeota [10, 11], also the distinct family D DNAP (PolD) [12–14]. In archaea that possess both DNAPs, it has been recently demonstrated that PolD, rather than PolB, is responsible for the synthesis of both DNA strands [15–18]. The structure of PolD has been recently solved, resulting in a surprising discovery that the catalytic core of PolD is homologous to that of the large subunits of the DNA-directed RNA polymerases (RNAPs) that are responsible for transcription in all three domains of life and many large DNA viruses [19–21]. These findings seem to shed unexpected light on the evolution of the replication machineries in the three domains of life as well as viruses. They might even help to infer the nature of the replication machinery in the LUCA suggesting an evolutionary scenario in which PolD takes the central stage as the ancestral replicative polymerase. In the rest of this article, we discuss the reasoning behind this scenario and its implications.

**Table 1:** DNA and RNA polymerases involved in replication and transcription in cells and viruses

Polymerase family	Core catalytic domain	Organisms	Functions	DNA-RNA switches
DNA-directed DNAPs				
PolA	Derived RRM-Palm	Bacteria, some phages, mitochondria	Repair in bacteria, replication in some phages, plant and fungal mitochondria, and mitochondrial plasmids	From DNAP to single-subunit RNAP in phages
PolB	RRM-Palm	Archaea, eukaryotes, some bacteria, many large DNA viruses	Replication in Crenarchaeota, eukaryotes, viruses; repair in other archaea, bacteria	To DNA template and product at origin(?)
PolC	Polβ-like nucleotidyl-transferase	Bacteria, a few phages	Replication	PolyA polymerase in eukaryotes
PolD	2xDPBB in a single protein	Archaea except for crenarchaea and some thaumarchaea	Replication	To DNA template and product at origin(?)
AEP	RRM-Palm	Archaea, eukaryotes, some bacteria, diverse mobile elements, many large DNA viruses	RNA priming of replication in archaea, eukaryotes, and dsDNA viruses; replication of plasmids and phages	From RNA synthesis during priming to DNA synthesis during plasmid replication
DNA-directed RNAPs				
Two-DPBB RNAP, two subunits	2xDPBB, one in each subunit	Bacteria, archaea, eukaryotes, many large DNA viruses	Transcription	To DNA template at origin(?) Viroid replication: to RNA template
Two-DPBB RNAP, single subunit	2xDPBB in a single protein	Many phages and linear cytoplasmic plasmids of fungi	Transcription	Eukaryotic RNAi: to RNA template
Single-subunit RNAPs	Derived RRM-Palm	Some phages, mitochondria	Transcription	None
RdRP-RT				
RdRP	RRM-Palm	RNA viruses	Replication	To DNA synthesis in RT(?)
RT	RRM-Palm	Retroelements, reverse-transcribing viruses	Replication in viruses and retroelements; telomere synthesis in eukaryotes	Using both RNA and DNA templates
RNAi eRdRP	2xDPBB in a single protein	Eukaryotes	Small RNA amplification	None

*AEP* archaeal-eukaryotic primase, *DNAP* DNA polymerase, *DPBB* double-psi beta-barrel, *RNAP* RNA polymerase, *RdRP* RNA-dependent RNA polymerase, *RRM* RNA recognition motif, *RT* reverse transcriptase

## Homologies among DNA and RNA polymerases

The homologous relationships among the DNA and RNA polymerases allow us to infer the evolution of the replication and transcription machineries in the 3 domains of life and in viruses. The key relationships are described below and summarized in Table 1 and Fig. 1.

### DNA and RNA polymerases with a catalytic core consisting of two double-psi beta-barrel domains

PolD contains two double-psi beta-barrel (DPBB) domains that both contribute catalytic residues to the DNAP active site. These two DPBB domains have homologous counterparts in the RNAP that is universally conserved in all 3 domains of life (Fig. 1a) and is also encoded by many large viruses with dsDNA genomes (Table 1) [19, 21–25]. In cellular RNAPs, the two DPBB domains reside in separate large subunits [26, 27]. Numerous large viruses with dsDNA genomes, including some tailed bacteriophages as well as nucleocytoplasmic large DNA viruses (NCLDV), such as the thoroughly characterized vaccinia virus and the giant mimiviruses, and baculo-like viruses of eukaryotes, also encode RNAPs with two DPBB domain-containing subunits [28–32]. Other viruses, for example, bacillus subtilis SPβ prophage and thermus thermophilus phage P23-45, encode single-subunit RNAPs that contain both DPBB domains within a single polypeptide and are highly divergent, with a limited sequence similarity to the cellular RNAPs [33, 34]. The eukaryotic RNA-dependent RNA polymerase (eRdRP, also known as QDE1 family, after the well-characterized representative from *Neurospora crassa*) that is responsible for small RNA amplification in eukaryotic RNA interference is another homolog of the RNAP catalytic subunits, in which the two DPBB domains are combined within the same protein (Fig. 1a) [27, 35]. Unexpectedly, the eRdRPs appear to be most closely related to a particular group of bacteriophage single subunit 2xDPBB RNAPs [27, 36]. The two large RNAP subunits are also fused in linear cytoplasmic plasmids from plants and fungi [37], suggesting that such fusions occur repeatedly in the course of RNAP evolution.

### Polymerases with a catalytic core based on the RRM domain

PolBs are also represented in all archaea [12, 38] but appear to function as the main replicative polymerases only in Crenarchaeota [39]. Multiple PolB paralogs are fully responsible for DNA replication in eukaryotes [40]. Additionally, PolBs are encoded by numerous viruses and some non-viral mobile genetic elements from all 3 domains of life [9, 29, 41–43]. Some bacteria encode PolBs of apparent virus origin that are involved in repair functions [44]. The catalytic core of PolB is the RNA recognition motif (RRM) domain (also known as the Palm domain in the case of polymerases) that is unrelated to DPBB but is homologous to the core catalytic domains of reverse transcriptases (RT) and viral RNA-dependent RNA polymerases (RdRP) (Fig. 1b) [8, 45].

### Conserved C-terminal domains in archaeal and eukaryotic replicative DNA polymerases

The archaeal and eukaryotic replicative DNAPs, respectively, PolD and PolB, share a homologous C-terminal domain (CTD), suggesting that this domain could be specifically important for replication [40, 46]. The CTD contains a distinct Zn-finger as well as the PCNA-binding “PIP motif” [47–52] and interacts with the small DNAP subunit in both archaea and eukaryotes via a conserved interface [20].

### Polymerases and nucleotidyltransferases with a Polβ family core domain

The catalytic core of PolC, the bacterial replicative DNAP, is a distant homolog of the catalytic domain of the Polβ family nucleotidyltransferases. This family includes eukaryotic repair DNAPs (Fig. 1c), polyA polymerases and a variety of small-molecule nucleotidyltransferases which are likely to represent the ancestral state of the family [53, 54]. Only a few, poorly characterized phages encode PolC homologs, presumably a late acquisition in virus evolution [9].

### DNA and RNA polymerases related to bacterial PolA

PolA, a distant homolog of PolB, is a bacterial repair polymerase. Some phages, Sputnik-like virophages (a group of eukaryotic viruses within the family *Lavidaviridae*), as well as mitochondria and chloroplasts employ PolA homologs as rDNAPs [55–58]. In addition, some phages (e.g., the thoroughly studied phage T7) encode distant homologs of PolA which function as single-subunit RNAPs [59]. These single-subunit phage RNAPs are the apparent ancestors of the mitochondrial RNAP in most eukaryotes [60], with the exception of jacobids that retain the multisubunit, DPBB-based RNAP [61].

An additional important assumption is the RNA world hypothesis [62, 63]. Specifically, we assume a stage in the evolution of life, subsequent to the RNA-only era, when the replicating genomes of the protocellular life forms consisted of RNA including mRNA translated into proteins including RdRPs [64, 65]. We assume that translation evolved within the RNA world, giving rise to an RNA-protein world that presaged the advent of DNA as the dedicated information carrier and the DNA replication machinery, probably, via a reverse transcription stage.

## Origins of DNA replication and transcription

**Fig. 2.**
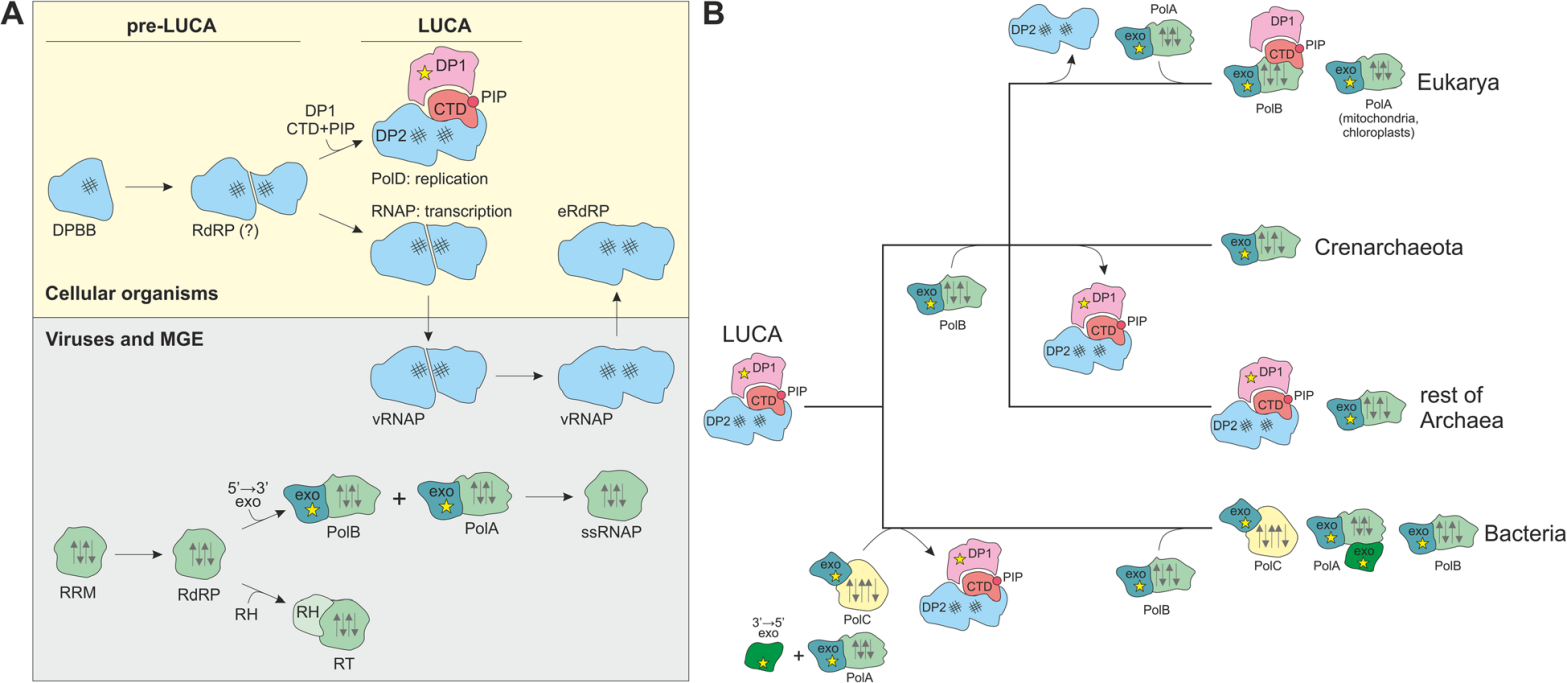
Proposed scenario for the origin and early evolution of DNA replication and transcription. **a** Evolution of cellular (top) and viral (bottom) polymerases from a double-psi beta-barrel (DPBB) and RNA recognition motif (RRM)-containing proteins, respectively. The first DPBB- and RRM-based polymerases have likely originated in protocells at the earliest stages of evolution, preceding the emergence of the Last Universal Cellular Ancestor (pre-LUCA); polymerases responsible for LUCA’s genome replication and transcription evolved from a common ancestor. DPBB-based RNAPs were exchanged between the cellular and viral worlds in both directions. **b** Scenario for the evolution of DNA replication machineries in the 3 domains of life. The multiple forms of PolB that are present in both archaea and eukaryotes are not shown for the sake of simplicity. Different domains and subunits are indicated with various shapes and colors. Yellow star indicates an active exonuclease domain. Note that DP1 subunit in the eukaryotic DNAPs is an inactivated exonuclease. DPBB is indicated with a triple hashtag symbol, whereas palm (RRM) domains are indicated with arrows. (e)RdRP, (eukaryotic) RNA-dependent RNA polymerase; (ss)RNAP, (single-subunit) DNA-dependent RNA polymerase; RT, reverse transcriptase; PolA, B, C, and D, DNA polymerases of families A, B, C, and D; DP1, small subunit of PolD with exonuclease activity; DP2, large subunit of PolD with DNA polymerase activity; RH, ribonuclease H domain; exo, exonuclease domain; CTD, C-terminal domain; PIP, PCNA-interacting motif; MGE, mobile genetic elements

The evolutionary connections among the polymerases of cellular organisms and viruses can be superimposed over the evolutionary tree of life that is based on the phylogenies of the universal proteins, namely, translation system components and the large RNAP subunits. This superposition suggests a plausible evolutionary scenario for the evolution of the replicative DNAPs that is intertwined with the evolution of RNAPs and RdRPs (Fig. 2). Given the ubiquity of the RNAPs with two DPBB-containing subunits in all 3 domains of life, this enzyme, obviously, predates the LUCA (Fig. 2a). The extant RNAPs readily assume RdRP activity, as demonstrated by the apparent evolutionary derivation of eRdRP from the catalytic subunit of phage RNAP [27], the involvement of plant RNAP II in viroid replication [66, 67] and animal RNAP II in hepatitis delta virus replication [68], and experimental data on the ability of RNAPs to use RNA as a template in vitro under certain conditions, such as molecular crowding [69].

Thus, it appears likely that the ancestral DPBB polymerase was an RdRP that antedated the origin of DNA replication (i.e., the advent of DNA as the genetic material) [8, 21] and could have been at least one of the enzymes responsible for RNA replication (see below). Given that all extant polymerases in this lineage contain two DPBB domains, it appears most likely that the primordial replicative polymerase already possessed this characteristic pair of DPBB domains that both contribute essential amino acid residues to the catalytic site. These domains conceivably evolved via duplication of a single ancestral DPBB domain (single DPBB domains are present in a variety of metabolic enzymes [21]) and could have resided in either a single or in two subunits (Fig. 2a). The ancestral DPBB form that gave rise to the first protein RdRP might have started as a non-catalytic RNA-binding domain that functioned as a cofactor to ribozyme RdRPs, but following the duplication, evolved the polymerase activity and displaced the ribozyme. Notably, given the apparent origin of the DPBB fold from the so-called RIFT barrel found in such proteins as EF-Tu-like translation factors and ribosomal protein L3 [70], the DPBB-based replication and transcription machineries might be rooted in the translation apparatus which predated DNA genomes.

The origin of DNA-based cells involved differentiation of the primordial two-DPBB RdRP into two distinct lineages: (1) the first replicative DNAP homologous to the extant archaeal PolD and (2) RNAP responsible for transcription (Fig. 2a). The separation of the replication and transcription machineries could have been precipitated by the accretion of additional domains in both classes of enzymes. The emergence of a DNAP capable of processive DNA synthesis that is required for replication was enabled by the fusion of the DPBB polymerase with a Zn-finger-containing DNA-binding and PCNA-binding protein that gave rise to the CTD and a separate fusion to an RNA-binding KH domain that became the N-terminal domain of the DNAP [20]. The original function of the sliding clamp remains obscure, but given its essentiality in all three domains of life, it is highly likely that a PCNA-like sliding clamp was a component of the LUCA’s replisome. The conservation of the PIP motif in both PolD and eukaryotic PolB [52], indeed, strongly suggests that PCNA binding and utilization as the sliding clamp during replication are ancestral features. Accordingly, under this scenario, the replicative DNAP of LUCA was a DPBB-CTD enzyme that subsequently survived as PolD and retained its role in replication, in all archaea except for Crenarchaeota (Fig. 2b). Additionally, either already in LUCA or at an early stage of archaeal evolution, PolD acquired a distinct small subunit, a phosphoesterase that became the proofreading exonuclease [20, 71]. Assuming that the ancestral RdRP contained the two DPBB domains within a single polypeptide, in the transcription lineage, the ancestral two-DPBB enzyme split into the two subunits each of which captured multiple additional domains including a clamp unrelated to PCNA [21, 26]. An alternative possibility is the fusion of the two ancestral DPBB-containing subunits in PolD. The subsequent evolution of RNAPs involved multiple, independent secondary fusions in archaea and bacteria as well as one or more fusion events that gave rise to the DPBB-based single-subunit bacteriophage RNAPs, one of which was recruited for the eRdRP function in eukaryotic RNAi (Fig. 2a) [72].

Post-LUCA, at the point of divergence between archaea and bacteria, the ancestral DPBB-containing replicative DNAP was displaced by PolC in the bacterial lineage (Fig. 2b). The bacterial DNAP apparently originated from an ancestral Polβ family nucleotidyltransferase although high divergence obscures its specific ancestry. The evolution of archaea involved the acquisition of multiple B family DNAPs (Fig. 2b). Given the widespread of this DNAP family in viruses, the virus origin of archaeal PolBs appears most likely. Ultimately, given the conservation of the core RRM domain, which most likely originated in the RNA-protein world (Fig. 2a), PolB, conceivably, evolved within the pool of mobile genetic elements including primordial viruses that would parasitize on protocells even in the pre-LUCA era. Specifically, PolB could originate from the RT of primordial retroelements. PolBs were similarly acquired by several groups of bacteria, apparently, at later stages of evolution (Fig. 2b), and in these cases, clearly, from bacteriophages. Thus, the PolB line of descent seems to represent the second, after the DPBB line, evolutionary path from a primordial RNA-binding domain (i.e., RRM) to both RNA and DNA polymerases.

In most archaea, PolBs are not involved in replication but rather in repair-related functions. However, in Crenarchaeota, two paralogous PolB forms replaced the ancestral PolD as the replicative DNAPs. A similar displacement occurred at the onset of the evolution of eukaryotes. In this case, PolB apparently recombined with PolD, replacing the polymerase domain but retaining the CTD [40] (Fig. 2b). Subsequent duplications of PolB at the onset of the evolution of eukaryotes yielded DNAPs ε, δ, α, and ξ, the first two of which are responsible for replication. The evolution of PolB in eukaryotes also involved inactivation of the small exonuclease subunit (archaeal DP1) that retained a structural role. Conceivably, the exonuclease activity of the small subunit became dispensable in eukaryotes due to its functional redundancy with the exonuclease domain of the PolB which replaced the PolD large subunit (archaeal DP2) [71].

The transition from DNA to RNA synthesis occurred also in the evolution of the family A of DNAPs. The origin of PolA that is conserved in nearly all bacteria and clearly is ancestral in the bacterial domain remains uncertain. One possibility is that PolA was derived from an ancestral RRM polymerase, perhaps, in a virus, and then was captured by the bacterial ancestor. In bacteria, PolA is a repair enzyme that is not directly involved in replication, but it functions as the replicative polymerase in some viruses and in eukaryotic mitochondria. Notably, PolA was captured by a group of phages as a single-subunit RNAP and was subsequently recruited in the same capacity by eukaryotic mitochondria, in all likelihood, from a phage [73, 74]. Thus, recruitment of viral polymerases, which are often more catalytically efficient than cellular counterparts [75, 76], by cellular organisms appears to be a recurrent theme in evolution, with postulated replacement of PolD by PolB at the onset of eukaryotes being but one example (albeit one of major importance).

Finally, a notable case of switching from RNA to DNA synthesis is the family of archaeal-eukaryotic primases (AEP), another group of RRM (Palm) domain polymerases [77, 78]. The primary function of AEP appears to be the synthesis of RNA primers in archaea, eukaryotes, and many large viruses, such as the NCLDV and herpesviruses. However, many plasmids and other mobile genetic elements in prokaryotes apparently employ AEP (also known as PrimPol) as the replicative DNAP [79].

## Concluding remarks

The origin of DNA replication is one of the most enigmatic subjects in the reconstruction of the early stages in the evolution of life because the replicative DNAPs (as well as primases and the main helicases involved in replication) are not homologous among bacteria, archaea, and eukaryotes. Until recently, this lack of conservation of the key elements of the DNA replication machinery precluded reconstruction of the ancestral state, suggesting multiple origins for DNA replication and even the possibility that LUCA was an RNA-based cell [2, 5]. However, given the universal conservation of other components of the replication apparatus, such as PCNA (sliding clamp), clamp loader ATPase, and ssDNA-binding protein, along with the inferred relatively high complexity of LUCA, comparable to that of modern prokaryotes, such scenarios appear unlikely. The line of reasoning developed here, based primarily on the recently discovered evolutionary connection between PolD and the universally conserved RNAP, allows inference of the ancestral DNAP. Under this scenario, the first transcriptase (RNAP) and the first replicative DNAP evolved from a common ancestor that probably functioned as an RdRP. Thus, the replicative DNAP of the LUCA was the direct ancestor of the extant archaeal replicative DNAP, PolD. The proposed evolutionary scenario appears parsimonious in that the two key processes associated with the advent of DNA genomes, replication and transcription, derive from a common ancestor. An alternative candidate for the role of the replicative DNAP of LUCA potentially could be PolB. However, a PolB-centered scenario for the evolution of replication lacks the symmetry in the evolution of replication and transcription. Besides, PolB is the replicative DNAP only in Crenarchaeota, eukaryotes, and in diverse viruses infecting hosts in all three cellular domains which seem to be best compatible with an origin in viruses or mobile genetic elements.

The proposed scenario traces two lines of descent from primordial RNA-binding domains, DPBB and RRM, to RdRPs (RTs) to RNAPs and DNAPs (Fig. 2a). Among these evolutionary lineages, the DPBB one is associated with the evolution of cells and the RRM one, with the evolution of viruses and mobile genetic elements. The causes of such asymmetry between hosts and parasites remain enigmatic. A notable aspect of the emerging picture of the evolution of replication and transcription is the switch between RNA and DNA template and products that, clearly, occurred on multiple occasions in evolution. Although highly challenging, validation of the current evolutionary scenario by experimental reconstruction of ancestral forms of RNA and DNA polymerases does not seem to be out of the question.

## Data Availability

Not applicable.
